# Editorial: Circuits of Resident Immunity Regulating Tissue Adaptation and Organ Homeostasis

**DOI:** 10.3389/fimmu.2022.901110

**Published:** 2022-04-11

**Authors:** Claudia U. Duerr, Christoph S. N. Klose, Arthur Mortha

**Affiliations:** ^1^ Charité - Universitätsmedizin Berlin, Corporate Member of Freie Universität Berlin und Humboldt-Universität zu Berlin, Institut für Mikrobiologie, Infektionskrankheiten und Immunologie, Berlin, Germany; ^2^ Department of Immunology, University of Toronto, Toronto, ON, Canada

**Keywords:** tissue residency, innate lymphocyte cells (ILCs), T cells, homeostasis, barrier surfaces

Each organ in our body serves a determined purpose, follows a distinct development pathway, contains specialized tissue cells and uses unique mechanisms to sustain homeostasis. The disease and pathogens threatening our organs are as diverse as themselves and require distinctly organized responses by the immune system. Patrolling, tissue-resident immune cells populate each organ at defined ratios and support homeostasis, defense and repair. Classical αβ T cells, γδ T cells, invariant T cells, Natural Killer (NK) cells and innate lymphoid cells (ILCs) are effector lymphocytes that aid in this function through local interactions with the microenvironment. Cytokines, growth factors and receptor-ligand interactions play critical roles in this process and are compellingly summarized within this collection of articles centered around the circuits that mediate the adaptation of lymphocytes to their hosting organ and the challenges to ensure defense and homeostasis.

Sheikh and Abraham start discussing the role of the interleukin (IL)-7 receptor alpha chain, a cytokine receptor chain used to identify ILCs but also critical for the survival and development of ILCs. Their review beautifully covers the importance of the IL-7 receptor in ILC biology. In light of cytokines and other pathways of tissue adaptation, Parker and Ciofani discuss the regulatory pathways underlying the earliest event during the effector specification of γδ T cells in the thymus. This review summarizes how the fate of γδ T cell specification in the thymus shapes their later effector profile within the organ. Cong and Wei discuss the role of human and mouse NK cells in the lung and provide a detailed insight into their function during homeostasis, infection, and cancer. Rafei-Shamsabadi et al. review ILCs in allergic skin inflammation. After providing an overview about ILC subsets and plasticity, the authors outline the role of ILCs in atopic dermatitis and contact hypersensitivity. They further discuss the role of group 2 ILCs (ILC2s) in the pathogenesis of allergic skin diseases and close their article with an overarching concept on how these intriguing cells influence the contextual balance of type I and type II immune responses. Centered around skin inflammation, Polese et al. analyze the contribution of T cells, NKT cells and ILCs to the pathogenesis of psoriasis, emphasizing the unique and overlapping contributions of their effector functions at various stages of this disease. The microenvironmental impact on the phenotype and function of heart ILC2s is the topic of an original research article by Deng et al., who define ILC2s as the major innate lymphoid cell (ILC) population in the unique microenvironment of the heart. Using parabiosis, the authors elegantly show that heart ILC2s are readily present at early life, retain tissue-residency during the steady state and increased in a model of myocardial necroptosis implicating their adaptation to environmental stress. Becker et al. summarize recent findings on kidney ILCs during homeostasis and inflammation. Importantly, the authors highlight that kidney ILC2s constitute a permanent immune population in both mouse and human, posing as potential therapeutic target for reinstating. In addition to the innate immune response, adaptive immunity plays an indisputably important role at immunoprivileged locations. During cerebral *Toxoplasma gondii* infections, an intact immunoproteasome (IP) limits the cellular protein stress to ensure an effective T cells response. Deficiency in three key subunits of the IP results in impaired parasitic control as reported in this original research paper by French et al. Domingues et al. focus their review on ILC3s as sentinels and regulators of tissue homeostasis. They discuss the impact of diets, the microbiota, circadian rhythm and neuroimmune interactions on ILC3s biology and function. The interactions with the commensal microbiota, adaptive lymphocytes, and other immune systems are further reviewed. Essential regulators of ILCs comprise neural factors, which are the focus of Jakob et al. Originating from the composition of the peripheral nervous system and, in particular, the enteric nervous system, this article outlines neuro-immune crosstalk, including but not limited to ILCs and the gastrointestinal tract. Therapeutic applications of neuro-immune interactions, such as in inflammatory bowel disease and other chronic inflammatory diseases, are discussed as well. Within the final article of this collection, Murphy et al. provide a comprehensive review of human and mouse ILCs across all tissues, emphasizing their beneficial and detrimental functions during the steady state and organ specific pathologies and infections. The authors highlight the underlying disease mechanisms and each ILC subset’s selective role on cause and consequence.

Collectively, these articles excellently summarize current concepts and mechanisms underlying the adaptation of lymphocytes to support organ homeostasis and defense.

## Author Contributions

All authors listed have made a substantial, direct, and intellectual contribution to the work and approved it for publication.

## Conflict of Interest

The authors declare that the research was conducted in the absence of any commercial or financial relationships that could be construed as a potential conflict of interest.

## Publisher’s Note

All claims expressed in this article are solely those of the authors and do not necessarily represent those of their affiliated organizations, or those of the publisher, the editors and the reviewers. Any product that may be evaluated in this article, or claim that may be made by its manufacturer, is not guaranteed or endorsed by the publisher.

